# Lamina Cell Shape and Cell Wall Thickness Are Useful Indicators for Metal Tolerance—An Example in Bryophytes

**DOI:** 10.3390/plants10020274

**Published:** 2021-01-31

**Authors:** Katharina Petschinger, Wolfram Adlassnig, Marko S. Sabovljevic, Ingeborg Lang

**Affiliations:** 1 Cell Imaging and Ultrastructure Research, Faculty of Life Sciences, University of Vienna, Althanstrasse 14, A-1090 Vienna, Austria; kpetschinger@gmail.com (K.P.); wolfram.adlassnig@univie.ac.at (W.A.); 2 Institute of Botany and Botanical Garden, Faculty of Biology, University of Belgrade, Takovska 43, 11000 Belgrade, Serbia; marko@bio.bg.ac.rs; 3 Department of Functional and Evolutionary Ecology, Faculty of Life Sciences, University of Vienna, Althanstrasse 14, A-1090 Vienna, Austria

**Keywords:** bioindication, bryophytes, moss, zinc, iron, cell shape, particulate matter

## Abstract

Bryophytes are widely used to monitor air quality. Due to the lack of a cuticle, their cells can be compared to the roots of crop plants. This study aimed to test a hypothetical relation between metal tolerance and cell shape in biomonitoring mosses (*Hypnum cupressiforme*, *Pleurozium schreberi*, *Pseudoscleropodium purum*) and metal sensitive species (*Physcomitrium patens*, *Plagiomnium affine*). The tolerance experiments were conducted on leafy gametophytes exposed to solutions of ZnSO4, ZnCl_2_, and FeSO_4_ in graded concentrations of 1 M to 10^−8^ M. Plasmolysis in D-mannitol (0.8 M) was used as a viability measure. The selected species differed significantly in lamina cell shape, cell wall thickness, and metal tolerance. In those tested mosses, the lamina cell shape correlated significantly with the heavy metal tolerance, and we found differences for ZnSO4 and ZnCl_2_. Biomonitoring species with long and thin cells proved more tolerant than species with isodiametric cells. For the latter, “death zones” at intermediate metal concentrations were found upon exposure to ZnSO4. Species with a greater tolerance towards FeSO_4_ and ZnSO_4_ had thicker cell walls than less tolerant species. Hence, cell shape as a protoplast-to-wall ratio, in combination with cell wall thickness, could be a good marker for metal tolerance.

## 1. Introduction

Bryophytes, especially mosses, are widely used for biomonitoring in different environmental studies [1,2,3]. Similar to primary roots in seed plants, most bryophytes do not possess a cuticle. Their leaflets consist of a monolayer of cells. Thus, bryophytes indiscriminately collect nutrients and other substances from atmospheric, mainly wet deposits. Therefore, they are perfectly suitable organisms to monitor the overall exposure at a given locality over a prolonged time span. Additionally, most bryophyte species are physiologically active over the winter and continue to adsorb deposited elements all year long.

A common method to analyze the air quality is to measure particulate matter (PM)-values [4]. PM may include solid particles and liquid droplets found in the air. PM2.5 are fine inhalable particles with a diameter of up to 2.5 µM, and PM10 includes inhalable particles with a diameter of 10 µM and lower. These particles may contain hundreds of different chemicals [5,6], some of which may seriously affect the human and animal respiratory system [6], resulting in a need for constant PM surveillance.

In Austria, iron and zinc hold the largest proportions of all heavy metals in the PM_10_ and PM_2.5_ range [4,7]. Therefore, the focus is on these two metals as they also play an important political and environmental role regarding air quality monitoring by the Austrian government to ensure policy compliances by the European Union. Furthermore, mosses may exhibit differences in metal uptake behavior and tolerance with respect to the element [8,9]. However, biomonitoring usually considers widely distributed species within the geographic region of interest and neglects possible differences of the species in terms of tolerance levels to elements or compounds. 

The over 12,000 moss species are representing a broad morphological diversity. Furthermore, each species manifests itself as protonema, leafy or thallous gametophore, or sporophyte [10]. Here, we focus on the leaflets (lamina, [11]) of the gametophyte since these represent most of the total surface. In spite of a multitude of different lamina shapes, most moss leaflets are composed of a single cell layer. The leaflets may consist of quadrate, rectangular, oblong, fusiform, rhomboidal, hexagonal, linear, flexuous, or vermicular shaped cells of extremely different size, sometimes supplemented by a costa (“midrib”), aberrant cells at the base of the leaf, or by lamellae, papillae and mamillae increasing the leaf surface [11,12]. Usually, lamina cells are classified as parenchymatic (roundish, rectangular, or hexagonal) or prosencymatic (elongated and interleaved; [13]). In this approach, we used a simplified determination of lamina cell form comparing roundish or hexagonal shapes with rectangular or elongated rectangular ones.

Although habitat or life forms have been frequently discussed as predictors of metal tolerance in mosses [8,14,15], we are not aware of studies considering moss morphology or anatomy as related to heavy metal pollutions. The focus is not on molecular differences in cell wall composition across kingdoms, as this has been thoroughly discussed by Sarkar et al. [16] or Fangel et al. [17]. Here, the hypothesis is tested that cell size and/or cell shape is related to tolerance of certain metals in selected moss species. Species commonly used in biomonitoring and species that are not considered as suitable were selected. Comparison of the metal tolerance, therefore, contributes to quality assurance in the field of biomonitoring of heavy metals.

## 2. Results

### 2.1. Lamina Cell Measurements

**Figure 1:**
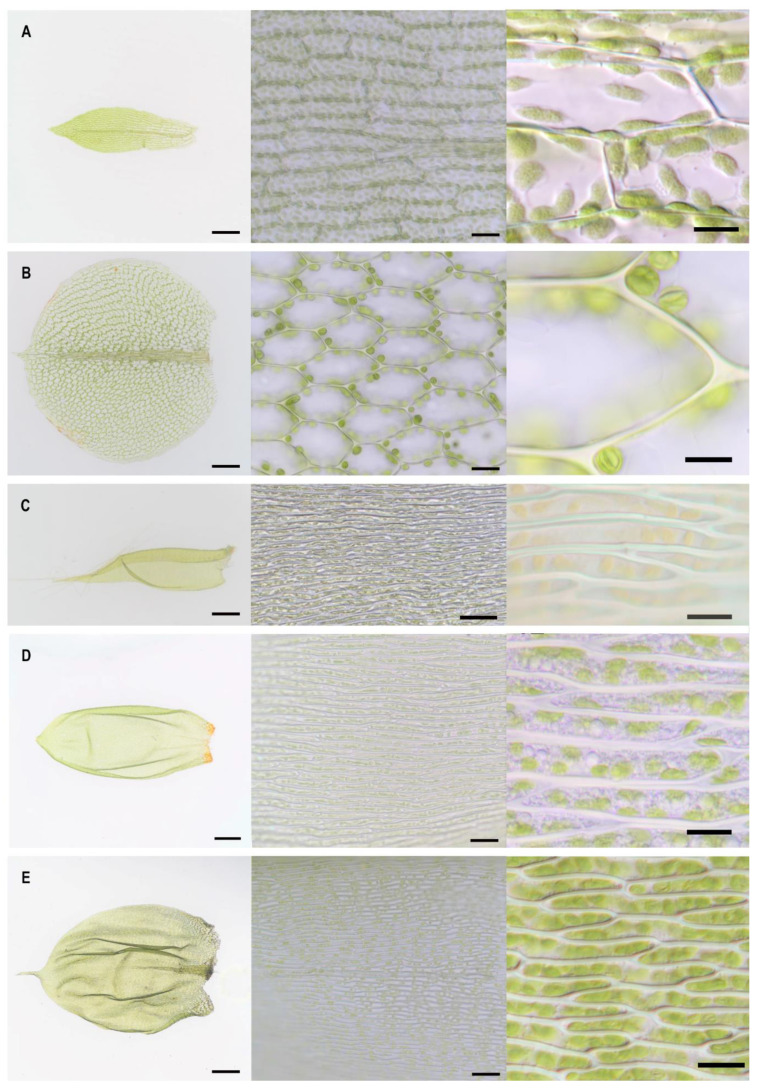
Lamina and cell shapes of five selected bryophyte species (**A**) *Physcomitrium patens*, (**B**) *Plagiomnium affine*, (**C**) *Hypnum cupressiforme*, (**D**) *Pleurozium schreberi*, and (**E**) *Pseudoscleropodium purum.* Bar: 250 μM for leaflet (overview); 25 μM for the respective cell shape (middle panel); 10 µM for close up (right panel).

The five different moss species (*Physcomitrium patens*, *Plagiomnium affine*, *Hypnum cupressiforme*, *Pleurozium schreberi*, and *Pseudoscleropodium purum*) have distinct leaflets and differ significantly in the size and shape of lamina cells (Figure 1, Table 1). Lamina cells showed a roughly rectangular shape for *P. patens* and a hexagonal shape for *P. affine*. *H. cupressiforme*, *P. schreberi*, and *P. purum* had elongated rectangular or linear lamina cells. No significant difference in cell shape was found within the same species. 

*P. schreberi* had the greatest average cell length (94 µM) followed by *H. cupressiforme* with 72 µM. The latter had the smallest cell width (3 µM), the biggest ratio of cell length to cell width (25), and the smallest cell area with only 220 µM^2^ (always respective mean values). The largest average cell width was measured in the moss *P. affine* (37 µM), but the largest cell area was found for *P. patens* (1979 µM^2^). With a value of 1, *P. affine* had the smallest ratio of cell length to cell width (Table 1).

**Figure 2:**
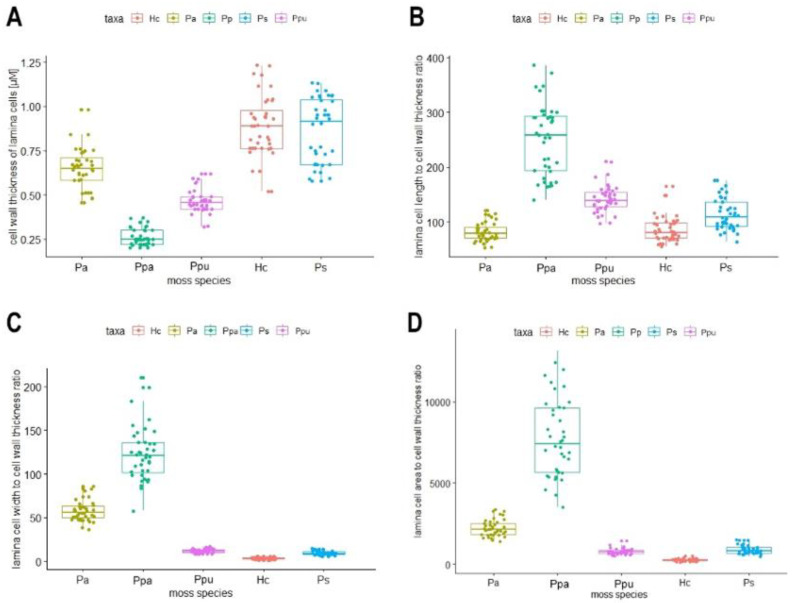
Box plots comparing the five investigated moss species. (**A**) cell wall thickness, (**B**) lamina cell length to cell wall thickness, (**C**) lamina cell width to cell wall thickness, and (**D**) lamina cell area to cell wall thickness. Pa: *Plagiomnium affine*, Ppa: *Physcomitrium patens*, Ppu: *Pseudoscleropodium purum*, Hc: *Hypnum cupressiforme*, and Ps: *Pleurozium schreberi.*

Cell wall thickness differed significantly between the five species (Dunn’s test: *p* < 0.05) except for *H. cupressiforme and P. schreberi* (*p* = 0.354). The thinnest cell walls were found in *P. patens* (µ = 0.26 µM). Wall thickness increased from *P. patens* < *P. purum* < *P. affine* < *H. cupressiforme* to *P. schreberi* with a mean thickness of almost 0.9 µM (Figure 2A). Thus, the biomonitor species *P. schreberi* and *H. cupressiforme* form thick cell walls compared to, e.g., *P. patens*. The tested species showed significant differences in the ratio of cell length to cell wall thickness (Figure 2B; Dunn’s text: *p* < 0.05) except for *P. affine* and *H. cupressiforme* (*p* = 0.3418) that both had a similar ratio of lengths to thick walls. This ratio increased from *P. affine* = *H. cupressiforme* < *P. schreberi* < *P. purum* < *P. patens* that had the short cells (µ = 63 µM) and thinnest walls (Figure 2B). Also, the ratio of cell width to cell wall thickness was significantly different in all tested species (Figure 2C; Dunn’s test: *p* < 0.05) and increased from *H. cupressiforme* < *P. schreberi* < *P. purum* < *P. affine* < *P. patens*. The thin cells of *H. cupressiforme* (µ = 3 µM) had a width to cell wall thickness ratio of four whereas *P. patens* with its wide cells (µ = 31 µM) had a ratio more than 100 times higher of cell width to cell wall thickness (Figure 2C). The ratio of the cell area to cell wall thickness was similar to the ratio of the width to wall thickness with the same increasing order of species (Figure 2D). The ratio of the cell area to cell wall thickness was 30 times higher in *P. patens* as compared to *H. cupressiforme*.

### 2.2. Metal Tolerance

**Figure 3:**
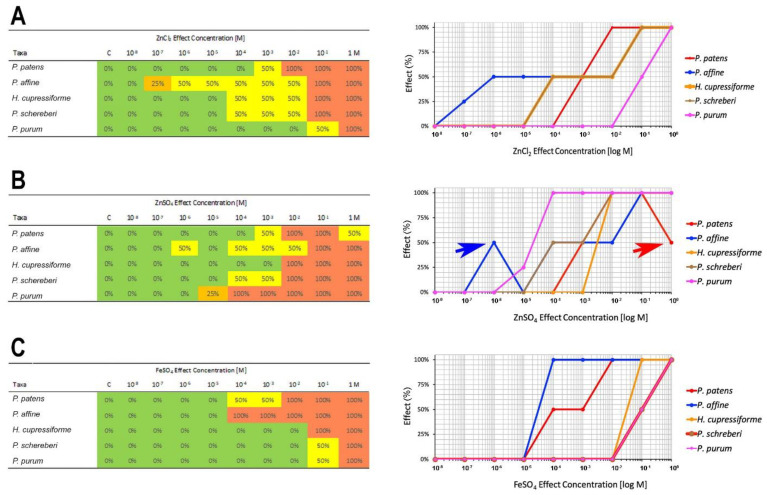
Percentage of dead lamina cells (0%, 25%, 50%, and 100%) and respective dose-response-curves for five moss species (*P. patens*, *P. affine*, *H. cupressiforme*, *P. schreberi*, *P. purum*) in tenfold dilution series of (**A**) ZnCl_2_, (**B**) ZnSO_4_ and (**C**) FeSO_4_. The arrows in B point to possible “death zones”; blue arrow: *P. affine*; red arrow: *P. patens*.

Metal tolerance was determined by viability tests using plasmolysis in 0.8 M mannitol (Figure 3). Living cells are able to undertake plasmolysis, whereas dead cells have lost semipermeable membrane function and therefore cannot plasmolyze [18]. The “no observed effective concentration” (NOEC) and “lowest observed effective concentration” (LOEC) for the three tested heavy metal solutions (ZnCl_2_, ZnSO_4_, and FeSO_4_) were assessed to achieve a numeric variable of tolerance data (Table 2). 

For each effect concentration, the median of the tolerance experiments was calculated (*n* = 40–80 cells per species). Since *P. affine* showed a death zone, the NOEC was formed by an average of three concentrations (10^−7^ M, 10^−6^ M, 10^−5^ M).

For ZnCl_2_, we observed a decreasing tolerance of moss species *P. purum* > *P. patens* > *P. schreberi* and *H. cupressiforme* > *P. affine*, whereby *P. affine* was the at least tolerant moss of the investigated species. *H. cupressiforme* and *P. schreberi* showed the same LOEC (10^−4^ M ZnCl_2_). *P. purum* could tolerate the highest observed concentration of 10^−2^ M ZnCl_2_ (Figure 3A). Apart from *H. cupressiforme* and *P. schreberi,* the tested species differ significantly with regard to their tolerance to ZnCl_2_ (*p* < 0.05). 

The tolerance of moss species to ZnSO_4_ dropped from *H. cupressiforme* > *P. patens* > *P. schreberi* > *P. purum* > *P. affine* (concentration range: 10^−8^–1 M) with *H. cupressiforme* showing the highest NOEC of 10^−3^ M (Figure 3B). There was a significant difference between all tested species to tolerate ZnSO_4_ (*p* < 0.05). Interestingly, death zones emerged in the case of *P. affine* showing 100 % viable cells at a concentration of 10^−5^ M ZnSO_4_ and only 50 % of viable cells at a lower concentration of 10^−6^ M ZnSO_4_. Additionally, a death zone is likely in *P. patens* between a concentration of >10^−1^ M ZnSO_4_: at 1 M ZnSO_4_, the tolerance tests showed only 50 % of dead cells, whereas, at a lower concentration (10^−1^ to 10^−2^ M ZnSO_4_), 100 % of cells died (Figure 3B, arrow).

Thus, visible effects of ZnCl_2_ (Figure 3A) could be observed at lower concentrations compared to ZnSO_4_ in *P. affine* and *H. cupressiforme* (Figure 3B) but not in *P. patens*, *P. schreberi*, and *P. purum*. Apparently, some mosses are more sensitive to ZnCl_2_ than to ZnSO_4_. *P. patens* and *P. schreberi* had the same LOEC for ZnCl_2_ and ZnSO_4,_ although the latter species with a slower transition and both tolerated a 10-fold higher concentration of ZnCl_2_ and ZnSO_4_ according to the percentage of viability. In contrast, *P. purum* survived a 10^4^ higher concentration of ZnCl_2_ than ZnSO_4_.

The overall tolerance to iron was greater than to zinc (Figure 3). In the concentration range of 10^−8^–1 M FeSO_4_, the tolerance of the tested species decreased as: *P. schreberi* = *P. purum* > *H. cupressiforme* > *P. patens* > *P. affine*. The species used for biomonitoring (*P. schreberi*, *P. purum* and *H. cupressiforme)* had the same LOEC of 10^−1^ FeSO_4_, whereas *P. patens* and *P. affine* had a LOEC of 10^−4^ M FeSO_4_. The transition from living to dead *P. affine* and *H. cupressiforme* cells was sudden when compared to the other species (Figure 3C).

### 2.3. Correlations between Cell Shape and Metal Tolerance

The NOEC and LOEC for the three tested heavy metal solutions (ZnCl_2_, ZnSO_4_, FeSO_4_, respectively) were assessed to achieve a numeric variable of tolerance data (Table 2) and to correlate them to cell shape. The results are shown in Table 3. 

A strong, negative correlation occurred between the cell width and the tolerance to FeSO_4_ (*ρ* = −0.85, *n* = 200, *p* ≤ 0.001, Table 3), and also the correlation between the ratio of the cell width to the cell wall thickness was highly negative (*ρ* = −0.85, *n* = 200, *p* ≤ 0.001). A strong, positive correlation between the ratio of the cell length to the cell width was also highly significant (*ρ* = 0.85, *n* = 200, *p* ≤ 0.001). The same trend, albeit with less strong correlation, was found for both zinc treatments. These data show an increased metal tolerance in species with elongated cells and thick walls.

## 3. Discussion

Many bryologists are aware of tolerance differences among selected species, their physiological state, life form or even genotypes when comparing or interpreting the results obtained. However, the mechanisms of resistance/tolerance to pollution substances remain obscured. Moreover, with such a huge variation of species, in addition to environmental, physiological, and morphological factors used in biomonitoring studies blur the pattern of pollutant tolerance. Therefore, we aimed to find a “simple” commodity like lamina cell shape that could be linked to metal tolerance.

In general, the monitoring of airborne heavy metal pollutants is a very difficult process [15]. Field receptor measurements are highly expensive, but they supply precise and reliable distribution-estimations about the airborne pollutants. However, they lack information on the effects of these pollutants on biological systems [19]. In this case, other methods were more appropriate, like the biomonitoring of heavy metal pollutants using bryophytes [1,2,15].

Different tolerance levels of the tested moss species to ZnCl_2_ and ZnSO_4_ were found but also to FeSO_4_ (Figure 3). Interspecific differences in the sensitivity were also reported by Tyler [20] as tested by net photosynthesis. We also used alternative tools and found different tolerance levels according to bryophyte species, life forms, or metal applied [9,18,21,22,23].

The tolerance experiments conducted with the biomonitoring mosses and the cultured mosses represent the tolerance of the species without considering their own background concentration of trace metals. In monitoring surveys, this background concentration of heavy metals in the mosses is usually not determined. However, if a pre-disposition with metals exits in the field samples, the tolerance levels would be lower, which leads to more conservative pollution estimations. The collection sites of the biomonitoring species used here are in the Viennese forest, away from civilization. No particular metal contamination is assumed. Furthermore, it should be noted that mosses have a relatively high, intrinsic concentration of zinc (about 20 μg/g), which is not due to emissions [24]. The same can be assumed for the tested iron samples. In *H. cupressiforme*, the comparison of field samples and tissue culture probes did not result in significant tolerance differences (A. Khan, unpublished data).

Death zones as found in this study for *P. patens* and *P. affine* exposed to ZnSO_4_ are known from the literature [25,26,27,28]. Biebl [25] reported death zones for certain bryophyte species, whereby a low and a high metal concentration resulted in little mortality, but intermediate metal concentrations were lethal. Biebl’s observations fit with the results of our study as a high (>1 M) concentration of ZnSO4 caused rather little mortality of lamina cells of *P. patens* (Figure 3). However, further studies are necessary to show if lamina cells of *P. patens* are completely viable at concentrations above 1 M ZnSO4. *P. affine* had a more distinct death zone at a lower concentration of 10^−6^ M ZnSO_4_. Url [28] also observed death zones in *Nardia scalaris* (a liverwort) for copper and vanadium. The physiological reasons for the death zones are still unclear and would need further investigations.

Metals are positively charged and become first adsorbed to cation exchange sites at the cell wall [29,30]. Sequential elution studies also found most metal retention in the cell wall [31,32]. Hence, metals are deposited to the apoplast, where they have little impact on the living cytoplasm. In mosses with thick cell walls, the apoplast contributes more to the total surface of the leaf compared to mosses with thinner walls. The same is true for species with elongated cells, compared to species with cells of the same volume but more globular or cube-shaped cells. If the cell wall plays a major role in metal retention [21], moss species with such cells or thick walls are therefore more tolerant.

In the present study, cell wall thickness was determined by light microscopy, but even at the highest possible resolution, the edges of cell walls may appear blurry (see Figure 2). To lower this statistical variance and conceive reliable results, a high number of measurements was performed (*n* = 40–80). Certainly, the molecular composition of the cell wall is also relevant as it has become adapted during evolution and in conquering various ecological niches [16,17]. However, bryophyte species used in biomonitoring appear to have a higher percentage of cell wall within the lamina. This renders them more tolerant, and therefore, they can adsorb more metals. The application of these species in biomonitoring thus results in higher metal amounts measured because other species, mosses or vascular plants, have thinner cell walls with less adsorption capacity. However, for the estimation of toxic pollution levels, we prefer a conservative approach that rather over-estimates the metal levels. This way, the present study confirms that the commonly used species are well suitable for biomonitoring.

## 4. Material and Methods

### 4.1. Plant Species

Five species of mosses were selected to study the lamina cell shape in combination with heavy metal tolerance. The chosen species are from five different families and four different orders. *Pleurozium schreberi* (Will. ex Brid.) Mitt. (Hylocomiaceae), *Hypnum cupressiforme* Hedw. (Hypnaceae), and *Pseudoscleropodium purum* (Hedw.) M. Fleisch. (Brachytheciaceae) are commonly used in environmental and biomonitoring studies and were collected in the forest near Vienna, Austria (14 March and 13 April, 2018). Additionally, we selected two species with very different cell shapes and sizes, *Physcomitrium* (*Physcomitrella) patens* (Hedw.) Bruch and Schimp. (Funariaceae) and *Plagiomnium affine* (Blandow ex Funck) T.J. Kop. (Mniaceae). They were cultured in a growth cabinet (Conviron) at 20 °C with a 14/10 h light/dark cycle. Sterile cultures of *P. patens* were propagated according to [33]. The widely used model species *P. patens* has a shorter life span than the bigger *P. affine*, and they also differ in life forms. None of the species shows a particular preference for metal contaminated sites, although *P. schreberi* has been found at the periphery of mine tailings [34,35]. Table 4 shows further details on the plant material and its cultivation.

### 4.2. Tolerance Tests

**Figure 4:**
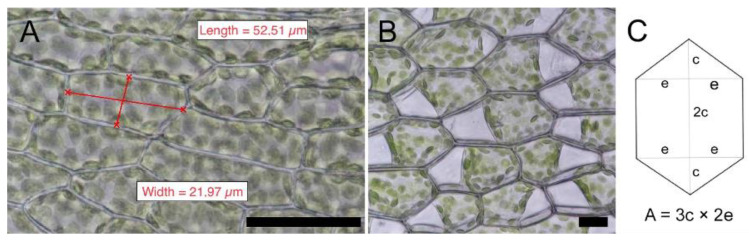
(**A**) measurements of cell length and widths in rectangular cells using the “extended focus” function (Nikon NIS -ElementsBR) of the microscope; bar: 50 µM; and (**B**) hexagonal cell type of *P. affine*, plasmolyzed in 0.8 M mannitol for 20 min; the protoplasts are detached from the cell wall; bar: 25 µM. (**C**) schematic and formula for area calculation of hexagonal cells (changed after Hnilica and Kohout 2018).

Two to three young but fully expanded leaflets of each moss species were placed in 96-well plates filled with serial dilutions (1 M to 10 nM) of ZnCl_2_ (Carl Roth, Karlsruhe, Germany), ZnSO_4_ (Merck), and FeSO_4_ (Merck), respectively. After 48 h, cell viability was tested via plasmolysis [18]; Figure 4B). In brief, after metal exposure, the leaflets were transferred into 0.8 M mannitol (Carl Roth, Germany) solution for 20 to 30 min. Then, the samples were placed in a droplet of the 0.8 M mannitol on a microscope slide, covered with a coverslip, and imaged in the light microscope (see below). The high sugar concentration of the mannitol solution causes osmotic water loss from the cell, mainly the vacuole. The water efflux from the cell eventually leads to a detachment of the protoplast from the cell wall as the vacuole diminishes in size (plasmolysis; Figure 4B). This process works by intact, semipermeable membranes only; damaged or dead cells, e. g. by high metal concentrations, do not plasmolyze. Hence, the plasmolytic viability tests allowed the determination of effect concentrations for the respective metal as well as the generation of dose-response curves. A minimum of 40 lamina cells were assessed per leaf. The leaflet was divided into four quarters; cells at the edges or midrib were not counted. In each quarter, the cells were analyzed individually using higher magnification, and the values summarized into 0%, 25%, 50%, or 100% dead cells, respectively, per quarter. Aiming to evaluate the significance of species differences in tolerance, the “no effect concentration” (NOEC) was analyzed in R Studio using Kruskal–Wallis rank sum test and Dunn’s test (see statistical analysis below). For the interpretation of the tolerance data, “the lowest observed effect concentration” (LOEC) was also used. 

### 4.3. Cell Measurements

Cell lengths were counted parallel to the longitudinal axis of the leaf, and cell width was defined as normally oriented to the longitudinal axis of the leaf (Figure 4). In all species, random measurements were done in fully developed leaves, i.e., leaf four and five from the top of the plantlets. Mid lamina cells are defined as those cells situated in the middle of the leaflets but not next to the margins and not next to the costa (“midrib”). This was done to reflect the cells that were covering the biggest leaf surface in each species and to avoid artifacts of different cell types present in some leaves. For cell wall measurements, at least two typical midleaf cells per lamina were randomly chosen, and at least 40 measurements per cell were performed towards all cell neighbors to reduce possible variabilities of wall thickness.

### 4.4. Microscopy

An upright light microscope (Nikon Eclipse Ni-U) was used in bright field and interference contrast mode. The instrument was equipped with the objectives Plan Fluor 4× (NA 0.13), Plan Apo 10× (NA 0.45), Plan Apo 20× (NA 0.75), Plan Fluor 40× (NA 0.75), Plan Fluor 60× oil immersion (NA 0.50–1.25), Plan Fluor 100x oil immersion (NA 1.30) and an attached camera (Nikon DS-Ri2). For picture processing, the software NIS-Element BR (Nikon), including an “extended focus” tool, was used. The calibrated measurements were directly exported to Excel (Microsoft Office 365). 

The cell areas of *P. patens*, *H. cupressiforme*, *P. schreberi*, and *P. purum* were calculated using the formula of a rectangle. For *P. affine*, we used the area of a hexagon [36] as it fitted best to the cell shape of this species (Figure 4C).

### 4.5. Statistical Analysis

Statistical analyses were conducted in Stata, version 14.2 (StataCorp LLC, College Station, TX, USA), and documented by archival of files containing all commands. Cell size was characterized by arithmetic mean (µ), standard deviation (SD), and sample size (n). Since samples differed significantly from a normal distribution, differences between the species were tested for significance by using the Kruskal–Wallis test with the post hoc Dunn’s test for pairwise comparison. Possible correlations between cell size and metal resistance were characterized by Spearman’s Rho (*ρ*). *ρ* > 0.8 was regarded as a strong, *ρ* > 0.5 as a moderate, and *ρ* > 0.2 as a weak correlation. *p* < 0.05 was regarded as significant throughout the study. Insignificant correlations were considered as meaningless, regardless of *ρ*. Figures were generated in R Studio, version 1.1.456 (RStudio, Boston, MA, USA).

## 5. Conclusions

In bryophytes, metal tolerance is species-specific, but the reasons for the different tolerance levels are not clear. Our data confirm a hypothetic relation of lamina cell shape and metal tolerance in the tested mosses. Those species with long and thin lamina cells cope better with high levels of metal than species with isodiametric cells. In the tested species, this correlation is particularly strong for iron, but a similar trend is shown for zinc. Apart from the cell shape, the thickness of the cell wall plays an important role in metal tolerance, most likely due to its adsorption capacity for positively charged ions. Although more bryophyte species should be tested in the future, plant cell anatomy, as in the case of lamina cells described here, is a helpful tool to indicate the metal tolerance of a moss.

## Figures and Tables

**Table 1:** Five moss species (*Plagiomnium affine*, *Physcomitrium patens*, *Pseudoscleropodium purum*, *Hypnum cupressiforme*, and *Pleurozium schreberi*) were compared by mean cell length (μm), cell width (μm), length to width ratio, shape, and mean cell area (μM^2^) of mid lamina cells (*n* = 40). SD = Standard deviation; µ = mean value; *p* = 0.0001 (Kruskal–Wallis test comparing all five species).

Moss Species	µ Cell Length (SD) (µM)	µ Cell Width (SD) (µM)	µ Cell Length to Width Ratio	Shape of Mid Lamina Cells	µ Cell Area (SD) (µM^2^)	µ Cell Wall Thickness (SD) (µM)
*Plagiomnium affine*	51 (4)	37 (3)	1	hexagonal	1402 (150)	0.65 (0.12)
*Physcomitrium patens*	63 (12)	31 (6)	2	rectangular	1979 (569)	0.26 (0.05)
*Pseudoscleropodium purum*	65 (8)	5 (1)	12	rectangular, longish	354 (73)	0.46 (0.07)
*Hypnum cupressiforme*	72 (11)	3 (1)	25	rectangular, longish	220 (53)	0.88 (0.17)
*Pleurozium schreberi*	94 (12)	8 (1)	12	rectangular, longish	735 (140)	0.86 (0.18)

**Table 2:** Median of no effect concentration (NOEC) and lowest observed effect concentration (LOEC) of tested substances (ZnCl_2_, ZnSO_4_, FeSO_4_ in Mol) for five moss species. *n* = 40–80 cells.

		NOEC Median			LOEC Median	
Species	ZnCl_2_	ZnSO_4_	FeSO_4_	ZnCl_2_	ZnSO_4_	FeSO_4_
*P. patens*	10^−4^	10^−4^	10^−5^	10^−3^	10^−3^	10^−4^
*P. affine*	5.5 × 10^−8^	5.55 × 10^−6^	10^−5^	5.5 × 10^−7^	10^−6^	10^−4^
*H. cupressiforme*	10^−5^	10^−3^	10^−2^	10^−4^	10^−2^	10^−1^
*P. schreberi*	10^−5^	10^−5^	10^−2^	10^−4^	10^−4^	10^−1^
*P. purum*	5.5 × 10^−3^	5.5 × 10^−6^	10^−2^	10^−1^	5.5 × 10^−5^	10^−1^

**Table 3:** Spearman correlation between morphometric parameters and maximum no-effect concentrations, shown as *ρ* (*p*). Strong (*ρ* ≥ 0.8) and highly significant (*p* < 0.01) correlations are highlighted (bold). *n* = 200 cells.

	ZnCl_2_	ZnSO_4_	FeSO_4_
Cell Length	0.16 (0.023)	0.27 (<0.001)	0.60 (<0.001)
Cell Width	−0.34 (<0.001)	−0.24 (<0.001)	**−0.85 (<0.001)**
Ratio Length/Width	0.24 (<0.001)	0.36 (<0.001)	**0.85 (<0.001)**
Cell Wall Thickness	−0.57 (<0.001)	0.24 (0.001)	0.50 (<0.001)
Ratio Length/Cell Wall Thickness	0.68 (<0.001)	−0.06 (0.406)	−0.17 (0.017)
Ratio width/Cell Wall Thickness	0.09 (0.202)	−0.25 (<0.001)	**−0.85 (<0.001)**

**Table 4:** Taxonomy, origin, and culture of the plant material.

Species	Collection	Culture Conditions
*Plagiomnium affine* (Blandow ex Funck) T.J. Kop. (Mniaceae)	laboratory	Non-sterile culture 20 °C, 14/10 h day/night
*Physcomitrium patens* (Hedw.) Bruch and Schimp. (Funariaceae)	laboratory	sterile culture 20 °C, 14/10 h day/night
*Pseudoscleropodium purum* (Hedw.) M. Fleisch (Brachytheciaceae)	48.183470, 16.067139	Natural habitat
*Hypnum cupressiforme* Hedw. (Hypnaceae)	48.183470, 16.044465	Natural habitat
*Pleurozium schreberi* (Will. ex Brid.) Mitt. (Hylocomiaceae)	48.183470, 16.066399	Natural habitat

## Data Availability

Data are available upon request.
